# Effective authentication of Placenta Hominis

**DOI:** 10.1186/s13020-018-0188-7

**Published:** 2018-06-18

**Authors:** Yat-Tung Lo, Mavis Hong-Yu Yik, Pang-Chui Shaw

**Affiliations:** Li Dak Sum Yip Yio Chin R & D Centre for Chinese Medicine, State Key Laboratory of Phytochemistry and Plant Resources in West China (CUHK) and School of Life Sciences, The Chinese University of Hong Kong, Shatin, N.T., Hong Kong, China

**Keywords:** Competitive ELISA, Diagnostic PCR, Human chorionic gonadotropin, Iodine test, Molecular authentication, Placenta Hominis, Pregnancy test strips, Progesterone

## Abstract

**Background:**

Human placenta is used to make the medicinal product Placenta Hominis in Asian countries. With its therapeutic benefits and limited supply, intentional or inadvertent adulteration is found in the market. In order to enforce the implementation of product description laws and protect customer rights, we established a hierarchical protocol involving morphological, chemical, biochemical and molecular diagnosis to authenticate this medicinal product.

**Methods:**

Ten samples claimed as Placenta Hominis were collected from herbal shops in China, Hong Kong and Taiwan. Species-specific diagnostic primers for human, cow, deer and sheep were designed for PCR amplification and subsequent DNA sequencing for species identification. Commercially available pregnancy test strip was used to detect human chorionic gonadotropin (hCG), and progesterone competitive ELISA kit was used to detect the presence of progesterone in samples. The presence of starch in samples was tested by adding small amount of iodine solution onto the samples.

**Results:**

Among the ten samples studied, results showed that no cow, deer and sheep DNA sequence was found in all samples. Five samples were genuine with the presence of human DNA, hCG and progesterone accompanied with the absence of starch fillers. On the other hand, four samples were adulterants which may be made from starch products. In addition, a sample was found as a mixture of Placenta Hominis and starch fillers, and it did not conform to the product requirement of Placenta Hominis.

**Conclusions:**

The comprehensive protocol developed involving morphological, chemical, biochemical and molecular diagnosis provides an accurate method to regulatory bodies and testing laboratories for the quality control of Placenta Hominis.

**Electronic supplementary material:**

The online version of this article (10.1186/s13020-018-0188-7) contains supplementary material, which is available to authorized users.

## Background

Placenta Hominis is a medicinal material made from human placenta. It is listed in Compendium of Materia Medica written by LI Shi-zhen in the sixteenth century. In this reference, Placenta Hominis has the action to warm the *kidney* and replenish *vital essence*, *qi* and blood. It is used to treat various diseases such as emaciation, hectic fever, night sweating, cough, anorexia, impotence, shortness of infertility and lack of lactation [1]. Nowadays, Placenta Hominis has been found to increase cell proliferation and metabolism [2], boost immune system [3] and it is used to treat bronchitis, asthma [4] and gastric ulcer [5]. Since human placenta is normally not available for trading, the material supply for making Placenta Hominis is limited and intentional or inadvertent adulteration is often found in the market. For instant, some dishonest merchants may use placenta from other mammals such as cow, deer and sheep, or even use starch products as adulterants [6].

Traditional methods to authenticate Placenta Hominis involve morphological and chemical approaches. However, variation in morphological appearance due to different preparation treatment and product form like powder [7] makes the morphological authentication difficult. Chemical authentication investigates the characteristic chemical markers in the medicinal materials. Thin layer chromatography (TLC) [8] and high-performance liquid chromatography (HPLC) [9] are the commonly used methods for identifying placenta, but they are not able to distinguish adulterants derived from placenta of other mammals as they share similar chemical components [10]. High-performance capillary electrophoresis (HPCE) has also been developed for authentication of Placenta Hominis from sheep’s placenta [11]. However, chemical constituents in individual placenta samples may vary [12] due to difference in physiological conditions, storage and processing methods.

Molecular authentication is especially suitable for animal-derived material since only few characteristic chemical compounds are present for chemical authentication when compared with plant-derived material. In addition, DNA markers are abundant, tissue independent with high resolution power [13]. For instance, DNA techniques have been employed to authenticate crocodile [14], snake species [15] and processed animal-derived concentrated Chinese medicine granules [16]. However, molecular techniques sometimes may not be applicable on processed products which have the DNA highly degraded or absent.

**Fig. 1:**
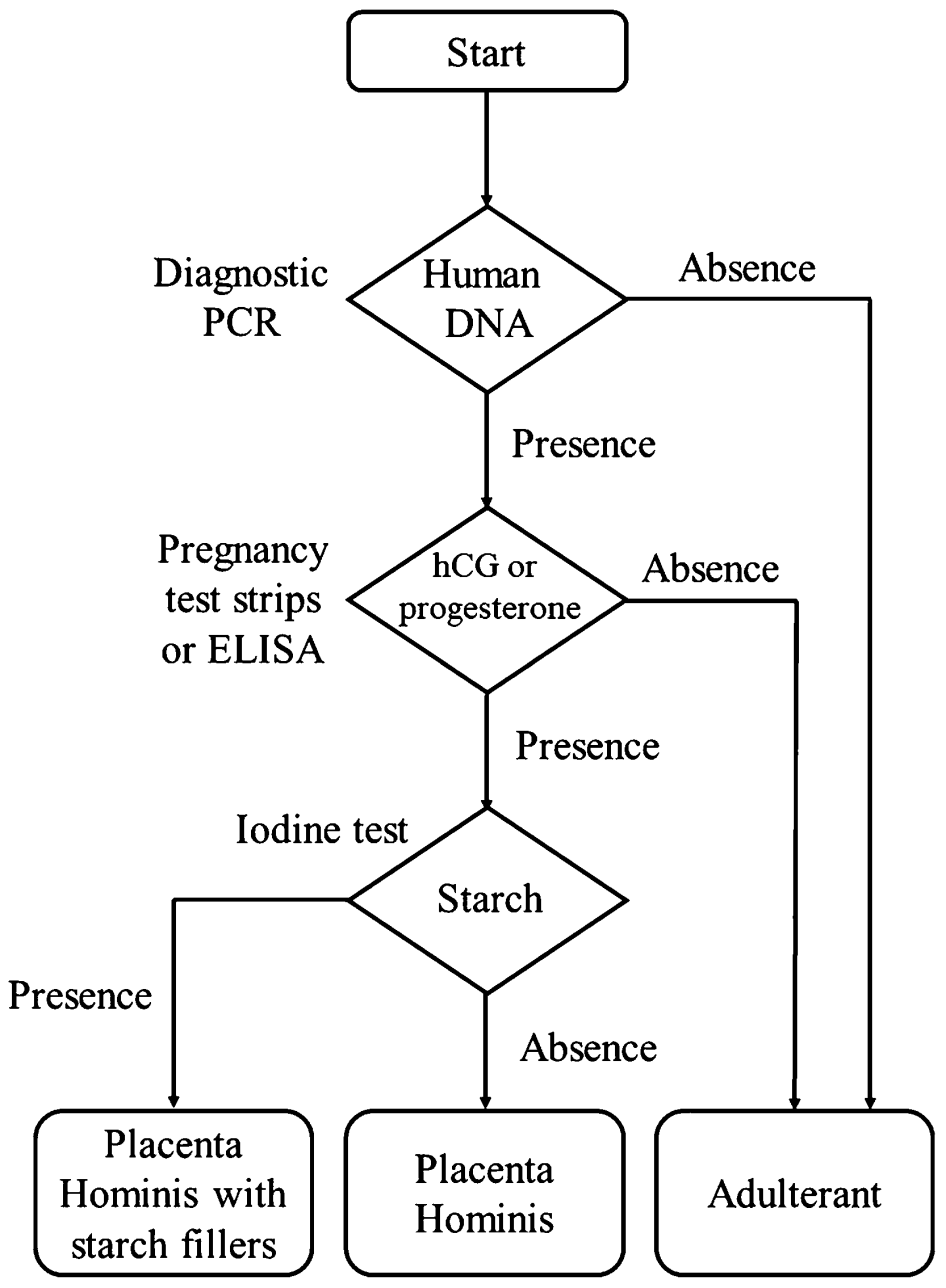
Suggested hierarchical protocol for the authentication of Placenta Hominis

Therefore, it is beneficial to employ a battery of independent methods to increase the accuracy of authentication. In this study, we have established a comprehensive protocol involving morphological, chemical, biochemical and molecular diagnosis (Fig. 1) for accurate and quick authentication of Placenta Hominis samples obtained from different places. A comprehensive protocol is needed for better implementation of Trade Descriptions Ordinance (Chapter 362) of Hong Kong and similar laws in other jurisdictions.

## Methods

The Minimum Standards of Reporting Checklist contains details of the experimental design, and statistics, and resources used in this study (Additional file 1).

### Sample studied

**Fig. 2:**
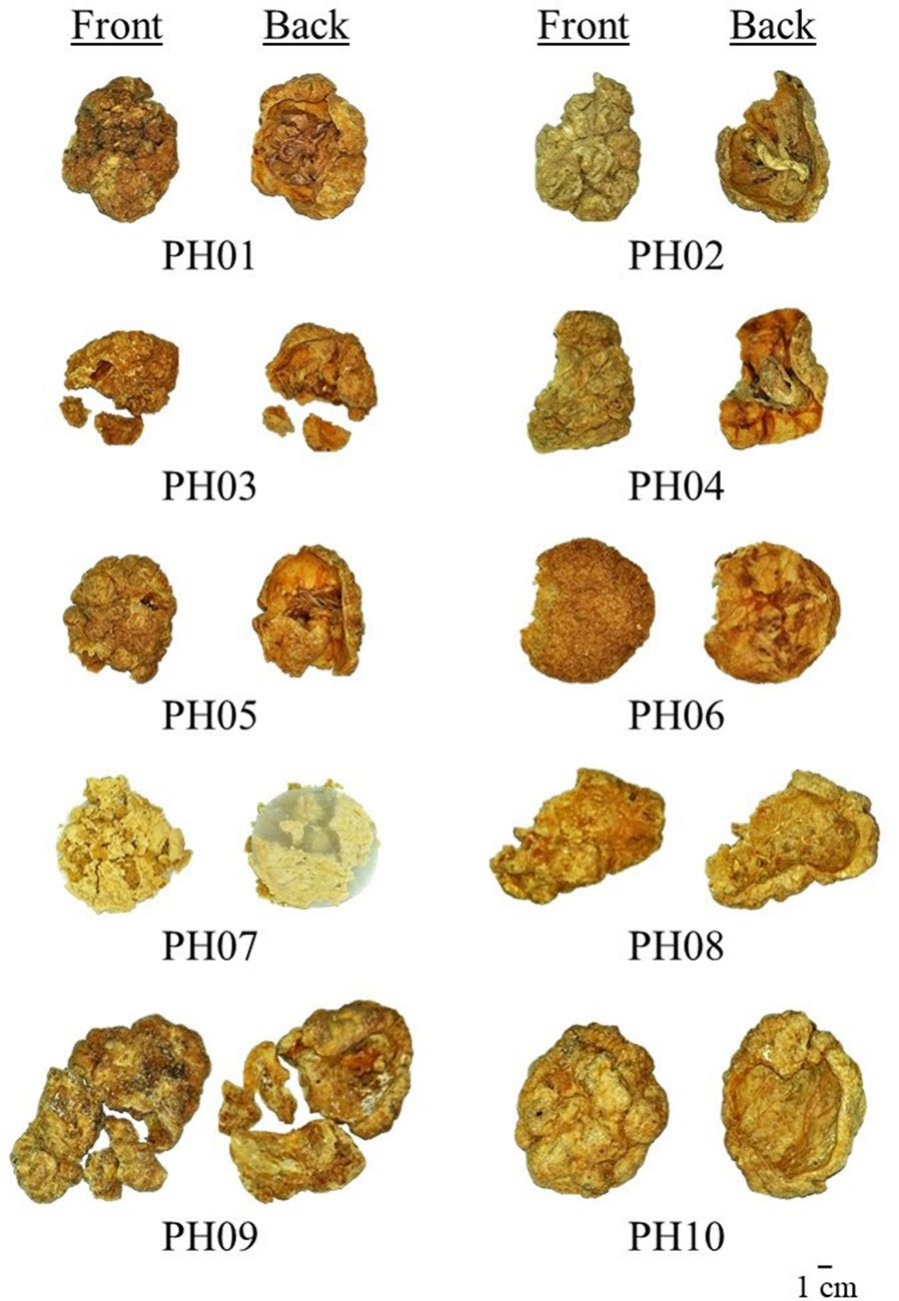
Images of Placenta Hominis samples studied

Ten samples claimed as Placenta Hominis were collected from herbal shops in China, Hong Kong and Taiwan. All specimens were deposited in the Institute of Chinese Medicine, The Chinese University of Hong Kong (Table 1). Images of the samples studied are shown in Fig. 2.

**Table 1:** Samples of Placenta Hominis studied

Sample code	Place of collection
PH01	Nanjing
PH02	Yunnan
PH03	Guangzhou
PH04	Guangzhou
PH05	Guangzhou
PH06	Taipei
PH07	Hong Kong
PH08	Yunnan
PH09	Guangzhou
PH10	Guangzhou

### Extraction of DNA and hormones

Each sample was washed with 70% ethanol and water to remove dusts and soils on the surface, followed by grinding into powder. For DNA extraction, 20 mg of the ground samples were extracted by following the manufacturer’s instruction in cell/tissue genomic DNA extraction kit (Biomed, Beijing, China) to obtain 50 µl DNA extract. For human chorionic gonadotropin (hCG) extraction, 300 mg of ground samples was first incubated in 1 ml of 10 mM phosphate-buffered saline (PBS) [137 mM NaCl, 2.7 mM KCl, 4.3 mM Na_2_HPO_4_, 1.47 mM KH_2_PO_4_, pH 7.4] for 3 h at 30 °C, followed by centrifugation at 604×*g* for 20 min to obtain the supernatant. For progesterone extraction, 300 mg of desiccated ground sample was shaken vigorously with 3 ml ethanol for 1 h in room temperature, followed by centrifugation at 1677×*g* for 15 min. The ethanol portion in the supernatant was then evaporated in a SpeedVac™ concentrator (Thermo Fisher Scientific, Waltham, MA, USA) and finally re-dissolved the extracted sample in 100 µl ethanol.

### Polymerase chain reaction (PCR) amplification and molecular analysis

DNA sequences of cytochrome c oxidase subunit I (COI) gene from human (*Homo sapiens*), cow (*Bos taurus*), deer (*Cervus elaphus* and *Cervus nippon*) and sheep (*Ovis aries*) were obtained from the GenBank database in National Center for Biotechnology Information (NCBI) and aligned using BioEdit 7 software. Species-specific diagnostic primers to differentiate each species were designed according to the polymorphic sites (Additional file 2) and shown in Table 2.

**Table 2:** Primers for amplification and sequencing

Species specificity	Primer name	Sequence (5′–3′)	Amplicon size (bp)	Annealing temp. (°C)
*Homo sapiens*	COI_human_F	GCAACCTTCTAGGTAACGACCAC	120	63
	COI_human_R	GGGGAACTAGTCAGTTGCCAAAG		
*Bos taurus*	COI_cow_F	CGACCAAATCTACAACGTAG	102	61
	COI_cow_R	GGAACAAGTCAGTTACCG		
*Cervus elaphus* and *Cervus nippon*	COI_deer_F	CTGCTTGGAGATGACCAAATT	125	59
	COI_deer_R	CCAATTATTAGGGGAACTAGTCAA		
*Ovis aries*	COI_sheep_F	GGCAACTGACTAGTTCCT	117	59
	COI_sheep_R	CATAGAGGATGCTAGGAGTAAC		

PCR amplification was performed in a 25 μl of reaction mixture with 2.5 μl of 10X PCR buffer [75 mM Tris, pH 8.8, 20 mM (NH_4_)_2_SO_4_, 1.5 mM MgCl_2_, 0.01% Tween 20], 2 μl of 2.5 mM dNTP mixture (Biomed), 1 μl of each 10 μM species-specific diagnostic primers, 1 μl of DNA sample and 0.4 μl of 5 U/μl *Taq* polymerase. PCR was conducted using Veriti™ Thermal Cycler (Thermo Fisher Scientific). The PCR programme included 35 cycles of 94 °C for 30 s, indicated annealing temperature (Table 2) for 30 s and 72 °C for 1 min. The amplification product was analyzed on 1.5% TAE gel electrophoresis stained with SYBR Safe DNA Gel Stain (Thermo Fisher Scientific), purified by DNA gel purification kit (Biomed) and performed DNA sequencing (BGI, Hong Kong). The obtained sequences were performed Basic Local Alignment Search Tool (BLAST) against GenBank nucleotide database. Query sequences were identified to species level with top hit of similarity.

### Detection of hCG by pregnancy test strips

Home pregnancy test strip (Mannings, Hong Kong) was purchased from a local pharmaceutical store. Each of the 100 µl extracted sample in PBS was added to the adsorbent tip of the test strip. The solution was then absorbed across the whole test strip until pink band appeared at the C (Control) section which indicated the test strip was working properly. For positive results with hCG concentration more than or equal to 20 mlU/ml, pink bands should be appeared for both C and T (Test) sections. For negative results, only a single pink band appeared at the C section.

### Detection of progesterone by enzyme-linked immunosorbent assay (ELISA)

Progesterone competitive ELISA kit (Thermo Fisher Scientific) was used to detect the presence of progesterone in samples. 400 μl of 1X Assay Buffer in the ELISA kit was added to 100 μl of each extracted sample in ethanol. The solution was then diluted into 1000 times using the same 1X Assay Buffer and transferred in 50 μl aliquots to the antibody coated wells. Competitive ELISA was then performed followed the manufacturer’s instruction. Results were measured at 450 nm with microtiter plate reader (BioTek Instruments, Inc., Winooski, VT, USA).

### Iodine test

The presence of starch in samples was tested by adding small amount of iodine solution onto the samples. Samples with color changed from brown to blue indicated the presence of starch and were labelled as positive, while samples remained unchanged in brown color indicated the absence of starch and were labelled as negative.

## Results

### Morphological characterization

Morphologies of the samples are shown in Fig. 2. They were majorly divided into three groups. For samples PH01–PH05, the irregular round or oval shape of samples had diameter around 10 cm. The color was yellow-white or yellow-purple. The outer surface was rough and uneven with grooves. The inner surface was relatively smooth with the remnant of umbilical cord-like structure in the center. The texture was crispy and there was a smell of blood. For samples PH06–PH07, they were pale yellow in color with a sheet of paper at the bottom. Their texture was biscuit-like and brittle. For samples PH08–PH10, the color was yellow-white and the sizes were ranged from 13 to 18 cm, which were much larger than other samples. The texture was very hard and without the presence of umbilical cord-like structure.

### Authentication by diagnostic PCR

**Fig. 3:**
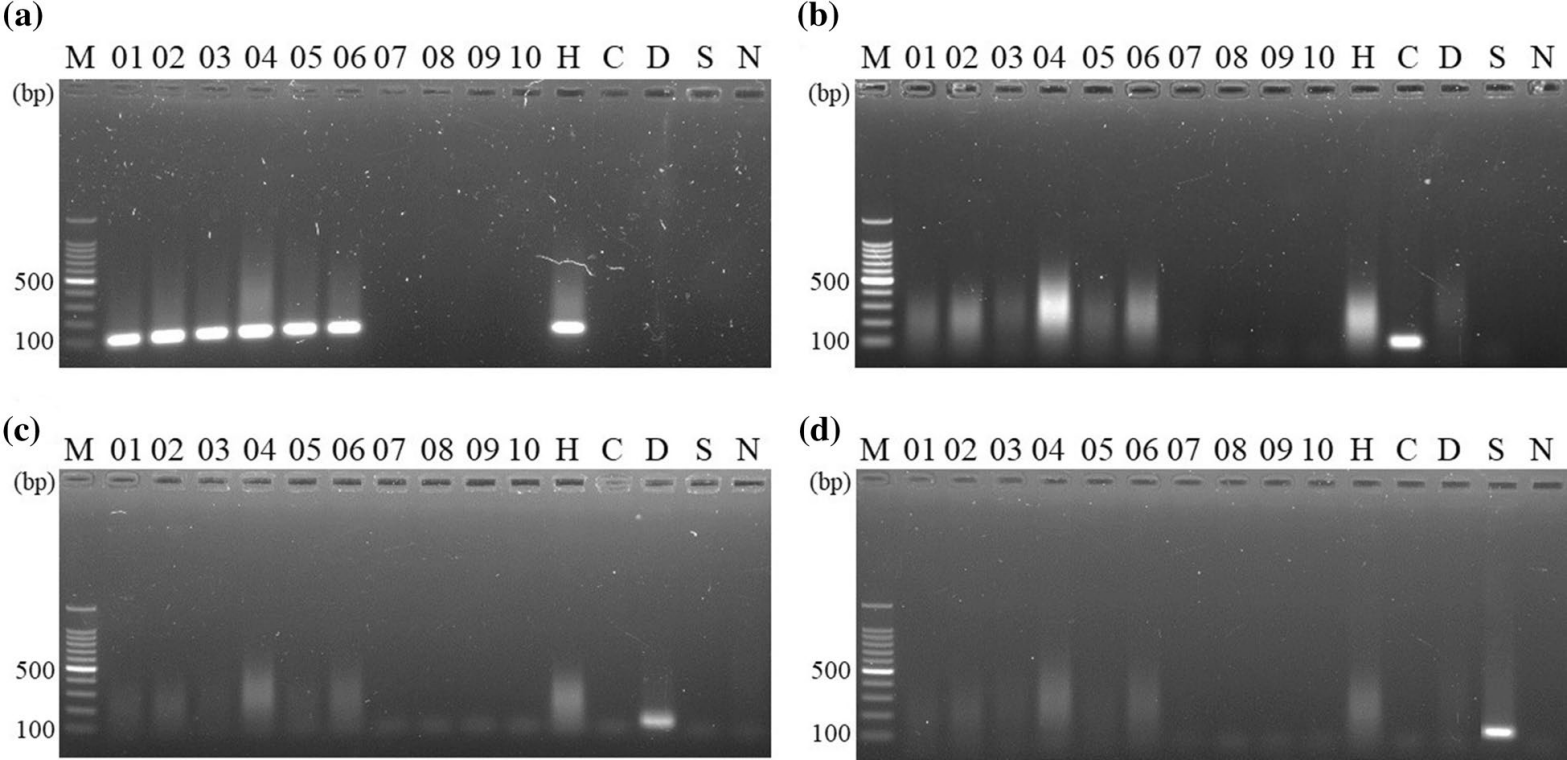
Diagnostic PCR using species-specific diagnostic primers for **a** human (*Homo sapiens*), **b** cow (*Bos taurus*), **c** deer (*Cervus elaphus* and *Cervus nippon*) and **d** sheep (*Ovis aries*). Lanes 01–10 represent PH01-PH10 samples, lanes H, C, D and S are positive controls with DNA from human, cow, deer and sheep, respectively. Lane M and N represent the DNA size ladder and negative control, respectively

For effective differentiation between *Homo sapiens* and other mammals which were commonly used as adulterants of Placenta Hominis, species-specific diagnostic primers were employed. With polymorphic sites at the primer 3’ end sequences (Additional file 2), only the concerned species could be amplified without non-specific PCR amplification for controls (Fig. 3). Using the human diagnostic PCR developed, PCR products with 120 bp were amplified from samples PH01–PH06 (Fig. 3a) with identity matched *Homo sapiens* perfectly (Additional file 3). In addition, no cow, deer and sheep DNA sequence was found in all samples (Fig. 3 and Table 3).

**Table 3:** Summary of iodine test, detection of hCG and progesterone hormones and diagnostic PCR results

Sample code	Iodine test^a^	HCG detection^b^	Progesterone conc.^c^ (ng/ml)	Diagnostic PCR^d^			
				Human	Cow	Deer	Sheep
PH01	N	Y	544.19 ± 120.76	Y	N	N	N
PH02	N	Y	435.17 ± 86.80	Y	N	N	N
PH03	N	Y	265.34 ± 49.59	Y	N	N	N
PH04	N	Y	461.48 ± 78.98	Y	N	N	N
PH05	N	Y	316.37 ± 52.92	Y	N	N	N
PH06	Y	Y	843.43 ± 93.75	Y	N	N	N
PH07	Y	N	Not detected	N	N	N	N
PH08	Y	N	Not detected	N	N	N	N
PH09	Y	N	Not detected	N	N	N	N
PH10	Y	N	Not detected	N	N	N	N

^a^In iodine test, Y and N indicate positive result with observable color change from brown to blue, and negative result without color change (i.e. brown), respectively

^b^In hCG detection, Y represents the presence of two pink bands at the Test (T) and Control (C) sections of pregnancy test strip, while N represents the presence of a single pink band at the C section only

^c^Assay buffer was set as blank and the data represent mean ± standard deviation (n = 3)

^d^In diagnostic PCR, Y and N represent the presence and absence of amplified products, respectively

### Authentication by hormone assays

**Fig. 4:**
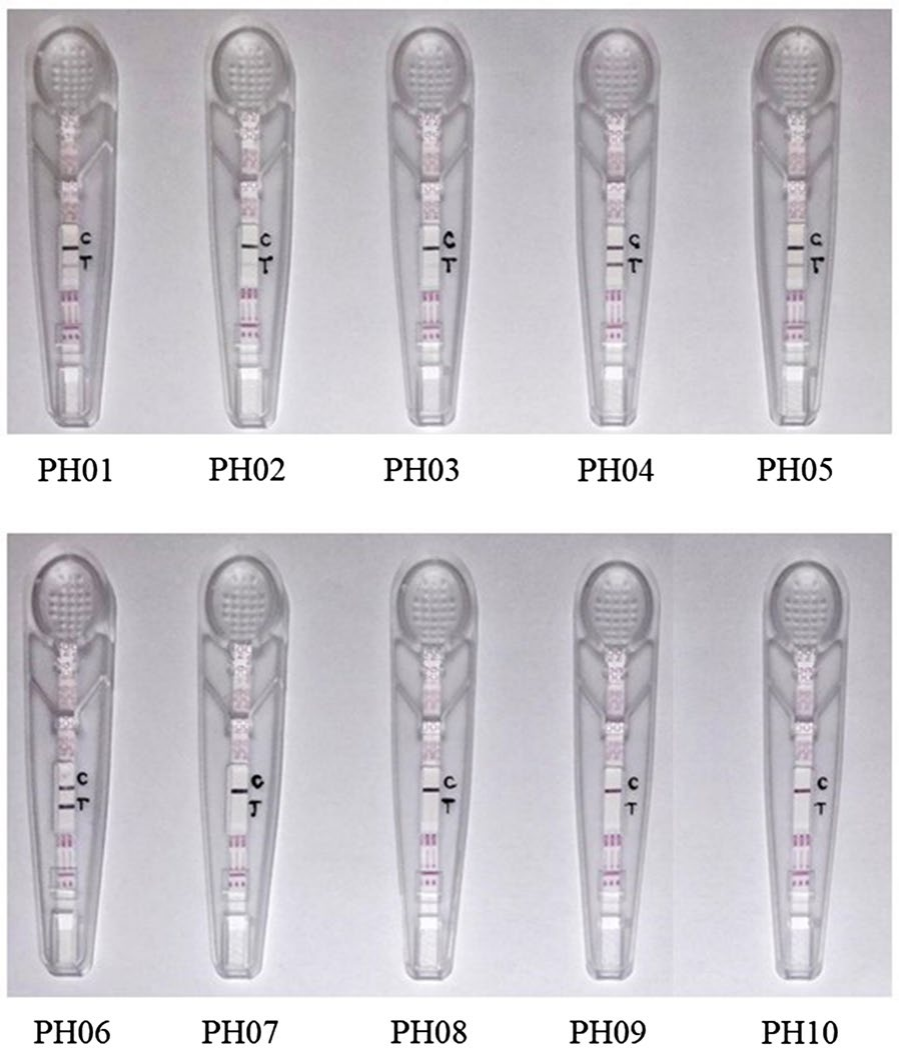
hCG test results showing the pregnancy test strips with pink bands presence/absence at the C (Control) and T (Test) sections. The presence of two pink bands at both C and T sections indicated the presence of hCG, while the presence of a single pink band at C section only indicated the absence of hCG

Commercially available pregnancy test strip was a rapid chromatographic immunoassay device for qualitative determination of hCG in samples with more than 99% accuracy of detection according to the manufacturer’s claim. The presence of two pink bands in the result window indicated the presence of hCG while one pink band in the C section indicated the absence of it. Results with no band in the C section should be regarded as invalid. In this study, hCG was detected in samples PH01–PH06 with two pink bands, while only one pink band appeared in the C section for samples PH07–PH10 (Fig. 4 and Table 3).

Placenta-derived progesterone in samples were detected by progesterone competitive ELISA kit. Progesterone level can also be quantified by comparing the absorbance reading at 450 nm with external standard curve. In this study, progesterone was found in samples PH01–PH06 with concentration ranged from 265 to 843 ng/ml. On the other hand, no progesterone above the detection limit of 47.9 pg/ml was detected in samples PH07–PH10 (Table 3).

### Authentication by iodine test

**Fig. 5:**
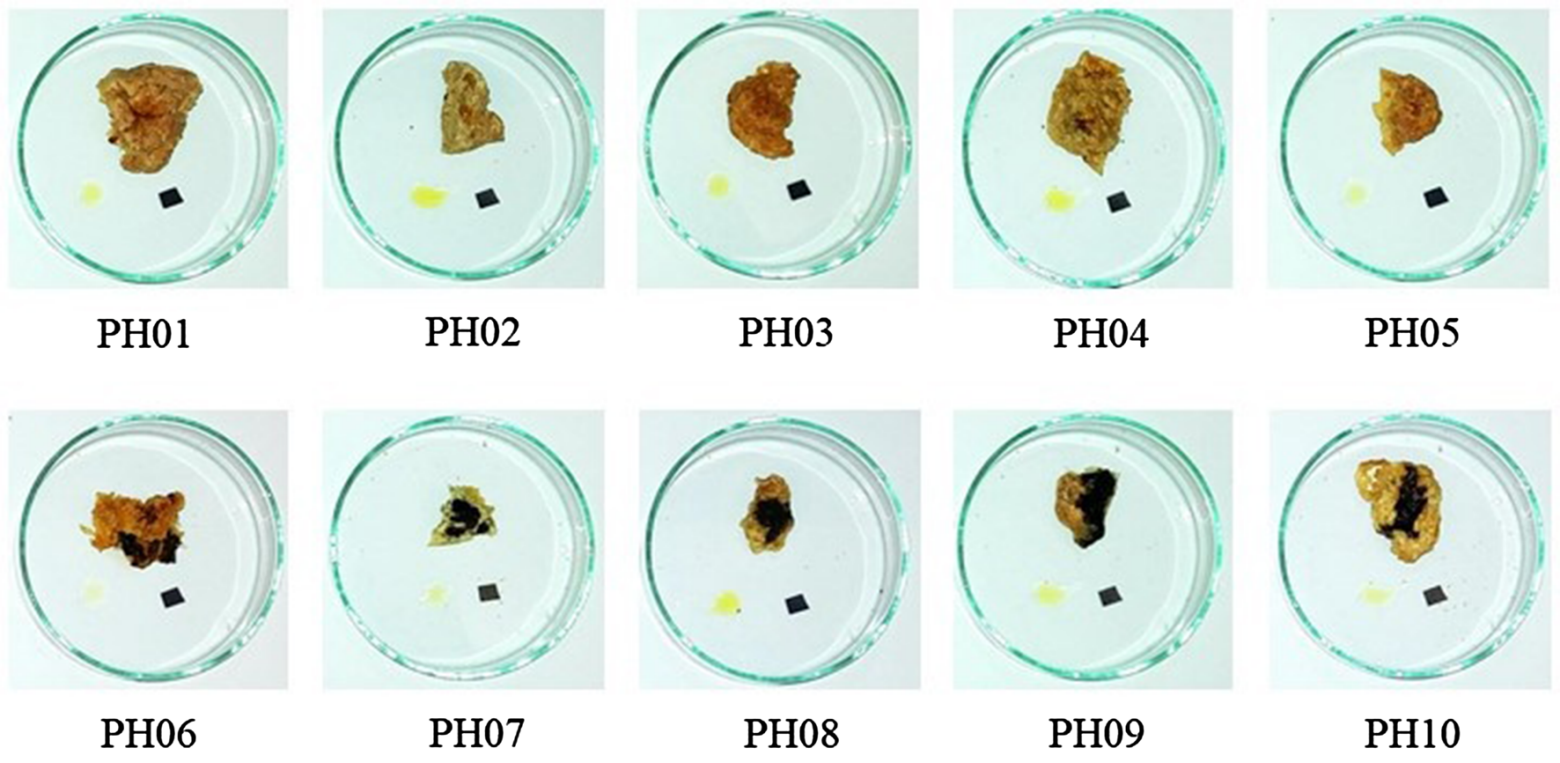
Iodine test results showing the color change for iodine solution added onto the sample with blue color indicated the presence of starch. Brown color (left) is negative control and blue color (right) is positive control

Presence of starch in sample was revealed by adding small amount of iodine solution onto the sample. The aqueous solution of the triiodide anion (I_3_^−^) from the iodine solution bound with starch molecule to form a very dark blue-black complex for visual determination. Results showed the presence of starch with dark blue-black color upon addition of iodine solution for samples PH06–PH10. On the other hand, the brown color of the iodine solution remained for samples PH01–PH05 which indicated the absence of starch in samples (Fig. 5 and Table 3).

## Discussion

In the present study, a comprehensive protocol was established to reveal the authenticity and quality of Placenta Hominis sold in market. According to the 2010 edition of Chinese Pharmacopoeia, genuine Placenta Hominis should be rounded or dish-shaped with 9–15 cm in diameter, yellow or yellowish-brown in color. One side of the material is uneven with irregular strips while another side is relatively smooth with remnant of umbilical cord in often. In this study, morphological appearance of samples PH01–PH05 match the above description.

The presence of human or other mammalian tissue was determined by using species-specific diagnostic primers for PCR amplification. In the preparation of Placenta Hominis, human placenta was steamed or boiled in water for a period of time, DNA in the samples was thus somewhat degraded. When designing the primers for diagnostic PCR, the amplicon to be amplified should keep as small as possible, while not compromising the differentiation power at species level, so as to reduce false negative results due to DNA fragmentation [16–19].

hCG is a glycoprotein produced by the human placenta after implantation [20, 21]. Progesterone is a steroid hormone involved in the female menstrual cycle, gestation and embryogenesis, and it is present in placenta [22, 23]. The presence of hCG and progesterone in the tested samples were indicated by commercially available pregnancy test strip and progesterone competitive ELISA kit, respectively. The positive results of pregnancy test strip indicated the hCG concentration was equal to or more than 20 mlU/ml. For sample PH03, the band intensity on the T section of the result window was lighter than others (Fig. 4). Nevertheless, it was still regarded as positive according to the manufacturer’s instruction. This sample also had a lower progesterone concentration (Table 3). These show that the quality of PH03 is inferior to other genuine samples.

In Placenta Hominis, starch should not be found. Samples PH07–PH10 had starch while without human DNA, hCG and progesterone (Table 3), implied that they were adulterants of Placenta Hominis and they may be made from starch products. An interesting finding is that for sample PH06, starch as well as human DNA, hCG and progesterone were found (Table 3). It is concluded that sample PH06 may be a mixture of Placenta Hominis and starch fillers. Traditionally, Placenta Hominis is produced from the whole human placenta only and thus the presence of starch in PH06 does not conform to the product requirement.

**Fig. 6:**
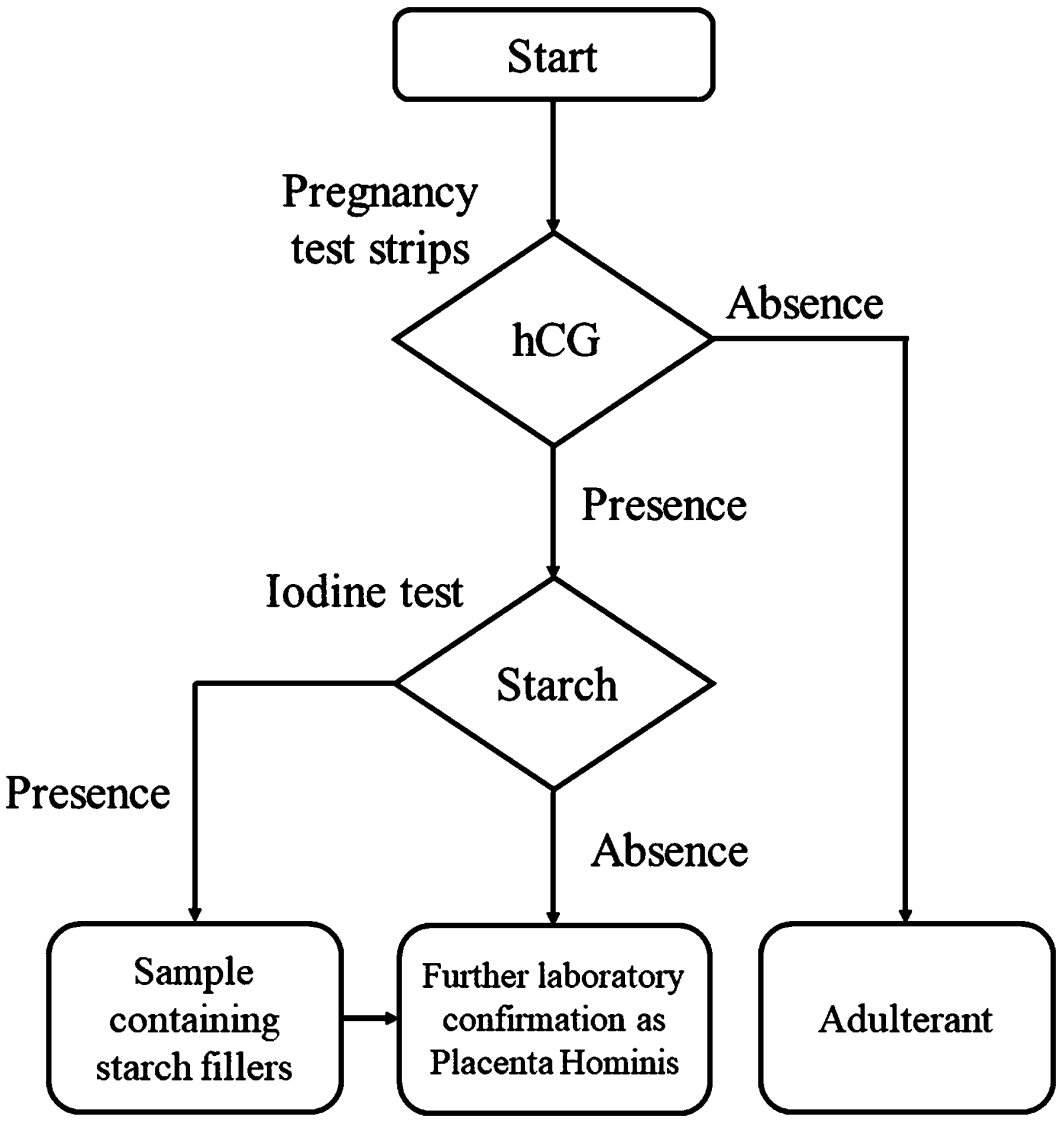
Suggested hierarchical protocol for preliminary identification of Placenta Hominis for Chinese medicine practitioners and customers without laboratory facilities

Establishing an objective method to identify Placenta Hominis is critical for the effective implementation of product description laws and safeguarding the consumer rights. In this study, a hierarchical protocol combining morphological, chemical, biochemical and molecular approach has been proposed to identify Placenta Hominis and assess its quality (Fig. 1). It is suggested that for Chinese medicine practitioners and customers without laboratory facilities, the authenticity of Placenta Hominis can be preliminary tested by observing morphological traits and using commercially available pregnancy test strips plus iodine test (Fig. 6). Diagnostic PCR and ELISA may be used for further confirmation. Our work showed that samples PH01–PH05 were genuine, with samples PH01, PH02, PH04 and PH05 of higher quality. On the other hand, samples PH07–PH10 were adulterants and sample PH06 was manufactured unconventionally.

## Conclusion

Our work has established a multi-disciplinary approach (morphological inspection, molecular identification, hormone assays and iodine test) for the authentication of Placenta Hominis and revealed that a substantial amount of this medicinal material in the market are adulterated.

## Additional files

**Additional file 1.** Minimum Standards of Reporting Checklist.

**Additional file 2.** Sequences alignment and species-specific diagnostic primer design.

**Additional file 3.** DNA sequence of the amplified COI gene from samples.

## Abbreviations

COI: Cytochrome c oxidase subunit I
DNA: Deoxyribonucleic acid
ELISA: Enzyme-linked immunosorbent assay
hCG: Human chorionic gonadotropin
HPCE: High-performance capillary electrophoresis
HPLC: High-performance liquid chromatography
NCBI: National Center for Biotechnology Information
PBS: Phosphate-buffered saline
PCR: Polymerase chain reaction
TLC: Thin layer chromatography

## References

[1] Li SZ, Luo XW (2003). Compendium of Materia Medica: Bencao Gangmu.

[2] Wang L (2001). The culture of the new small rat epidermal cells: the evaluation of effect for the human placental tissue extract on the cell’s physiological function. Chin Surfactant Deterg Cosmet.

[3] Cui YD, Cai YJ, Jin HK, Jin XJ, Jin SZ, Li YJ (2001). Study on immunological function of placental powder. Chin J Basic Med Trad Chin Med.

[4] Wu L, Li JX, Wang GL, Sun B, Xia C (2001). The evolution on pharmacological effect and clinic application of domestic animals and human placenta. Heilongjiang Bayi Nongken Daxue Xuebao.

[5] Zhuang JX (2002). Analysis of 40 cases in ulcer-curing of Placenta Hominis. Jiangxi J Trad Chin Med.

[6] Lu WC, Li YQ (2000). Beware of adulterants in Placenta Hominis powder. J Chin Med Med.

[7] Ou Yang CG (2004). The study on quality control of Placenta Hominis and its capsules. Chin J Info Trad Chin Med.

[8] Zhu ZX (1996). Authentication of Placenta Hominis and its adulterants by thin layer chromatography (TLC). Zhong Yao Cai.

[9] Zhu ZX (1996). Authentication of Placenta Hominis and its adulterants by UV light. Chin Trad Patent Med.

[10] Shi JY, Lü PY, Cheng JY, Liu YQ (1998). Studies of identification and quality to pangolin scales, Placenta Homins and their adulterants. J Shandong Univ Trad Chin Med.

[11] Gu J, Liu P, Li W (2006). Study on identification of Placenta Hominis and pseudo-placenta with high performance capillary electrophoresis. Acad J PLA Postgrad Med Sch.

[12] Zhang LB, Qiao CZ (1994). Comparison of progesterone, HCG and HPL contents in Placenta Hominis, other mammalian placentas and its processed products. Ti Erh Chun i Ta Hsueh Hsueh Pao.

[13] Mafra I, Ferreira IMPLVO, Oliveira MBPP (2008). Food authentication by PCR-based methods. Eur Food Res Technol.

[14] Yau FC, Wong KL, Wang J, But PP, Shaw PC (2002). Generation of a sequence characterized amplified region probe for authentication of crocodilian species. J Exp Zool.

[15] Wong KL, Wang J, But PP, Shaw PC (2004). Application of cytochrome b DNA sequences for the authentication of endangered snake species. Forensic Sci Int.

[16] Jiang LL, Lo YT, Chen WT, Shaw PC (2016). DNA authentication of animal-derived concentrated Chinese medicine granules. J Pharm Biomed Anal.

[17] Aslan O, Hamill RM, Sweeney T, Reardon W, Mullen AM (2009). Integrity of nuclear genomic deoxyribonucleic acid in cooked meat: implications for food traceability. J Anim Sci.

[18] Zimmermann J, Hajibabaei M, Blackburn DC, Hanken J, Cantin E, Posfai J, Evans TC (2008). DNA damage in preserved specimens and tissue samples: a molecular assessment. Front Zool.

[19] Lo YT, Li M, Shaw PC (2015). Identification of constituent herbs in ginseng decoctions by DNA markers. Chin Med.

[20] Gregory JJ, Finlay JL (1999). Alpha-fetoprotein and beta-human chorionic gonadotropin: their clinical significance as tumour markers. Drugs.

[21] Cole LA (2009). New discoveries on the biology and detection of human chorionic gonadotropin. Reprod Biol Endocrinol.

[22] Conti M, Chang RJ, Jameson JL, De Groot LJ (2015). Folliculogenesis, ovulation, and luteogenesis. Endocrinology: adult and pediatric.

[23] Brucker MC, Likis FE, King TL, Brucker MC (2010). Steroid hormones. Pharmacology for women’s health.

