# Supplementary material for: Effective authentication of Placenta Hominis
Source: Chin Med. 2018 Jun 18;13:32. doi: 10.1186/s13020-018-0188-7 (PMC6007028; doi:10.1186/s13020-018-0188-7)
Supplement: Supplementary file 2 — Additional file 2. Sequences alignment and species-specific diagnostic primer design. [file 13020_2018_188_MOESM2_ESM.docx]

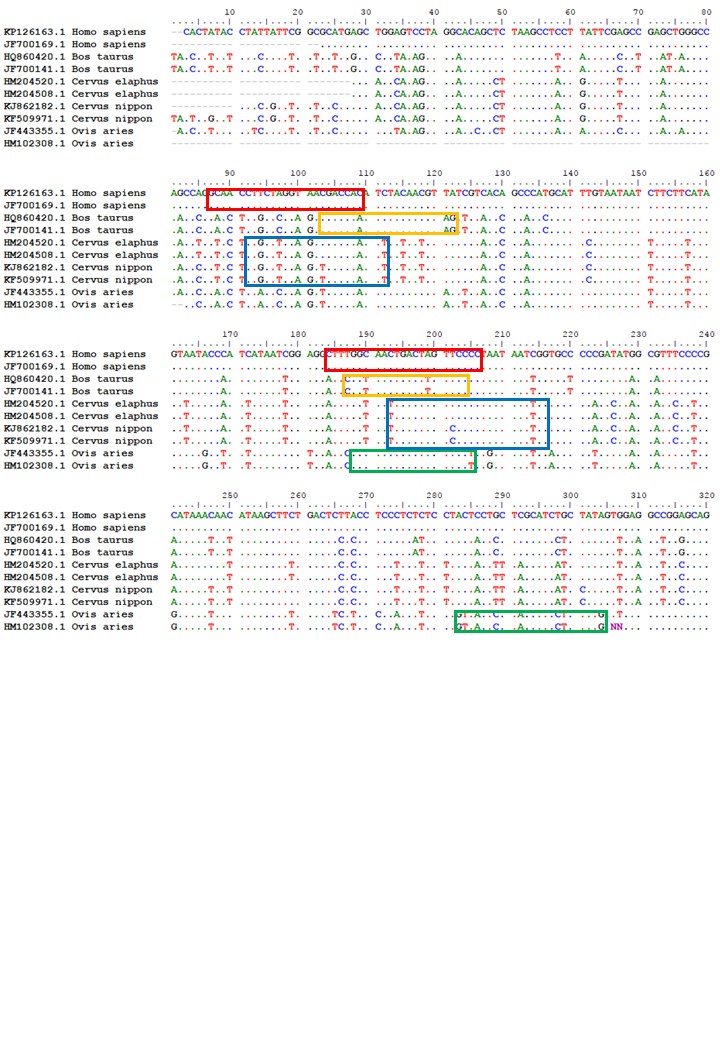


**Additional file 2 – Sequences alignment and species-specific diagnostic primer design.**

DNA sequence of COI gene from human (*Homo sapiens*), cow (*Bos taurus*), deer (*Cervus elaphus* and *Cervus nippon*) and sheep (*Ovis aries*) species were obtained from NCBI GenBank and compared with sequence alignment. Primers used in this studied were highlight in red, yellow, blue, and green boxes, respectively. Dots indicate the nucleotides identical to the top sequence (i.e. KP126163.1 *Homo sapiens*).
