# Supplementary material for: Effective authentication of Placenta Hominis
Source: Chin Med. 2018 Jun 18;13:32. doi: 10.1186/s13020-018-0188-7 (PMC6007028; doi:10.1186/s13020-018-0188-7)
Supplement: Supplementary file 3 — Additional file 3. DNA sequence of the amplified COI gene from samples. [file 13020_2018_188_MOESM3_ESM.docx]

**Additional file 3 – DNA sequence of the amplified COI gene from samples.**

The obtained sequences were BLAST against GenBank database and identified as the top hit species.

| **Sample code** | **Primer specificity** | **Size (bp)** | **DNA sequences (5’ - 3’)** | **Identity** | **% of similarity** |
| --- | --- | --- | --- | --- | --- |
| PH01 | Human | 74 | ATCTACAACG TTATCGTCAC AGCCCATGCA TTTGTAATAA TCTTCTTCAT  AGTAATACCC ATCATAATCG GAGG | *Homo sapiens* | 100% |
| PH02 | Human | 74 | ATCTACAACG TTATCGTCAC AGCCCATGCA TTTGTAATAA TCTTCTTCAT  AGTAATACCC ATCATAATCG GAGG | *Homo sapiens* | 100% |
| PH03 | Human | 74 | ATCTACAACG TTATCGTCAC AGCCCATGCA TTTGTAATAA TCTTCTTCAT  AGTAATACCC ATCATAATCG GAGG | *Homo sapiens* | 100% |
| PH04 | Human | 74 | ATCTACAACG TTATCGTCAC AGCCCATGCA TTTGTAATAA TCTTCTTCAT  AGTAATACCC ATCATAATCG GAGG | *Homo sapiens* | 100% |
| PH05 | Human | 74 | ATCTACAACG TTATCGTCAC AGCCCATGCA TTTGTAATAA TCTTCTTCAT  AGTAATACCC ATCATAATCG GAGG | *Homo sapiens* | 100% |
| PH06 | Human | 74 | ATCTACAACG TTATCGTCAC AGCCCATGCA TTTGTAATAA TCTTCTTCAT  AGTAATACCC ATCATAATCG GAGG | *Homo sapiens* | 100% |
